# Childbearing intentions in a low fertility context: the case of Romania

**DOI:** 10.1186/s41118-018-0046-6

**Published:** 2019-01-18

**Authors:** Alexandra-Andreea Ciritel, Alessandra De Rose, Maria Felice Arezzo

**Affiliations:** 1 Department of Social Statistics and Demography and ESRC Centre for Population Change, Social Sciences University of Southampton, University Road, Southampton, SO17 1BJ12 UK; 2 Department of Methods and Models for Economics, Territory and Finance – Sapienza University of Rome, via del Castro Laurenziano, 9, 00161 Rome, Italy

**Keywords:** Fertility intentions, Theory of planned behaviour, Gender and generation survey, Romania, Developmental regions

## Abstract

This paper applies the Theory of Planned Behaviour (TPB) to find out the predictors of fertility intentions in Romania, a low-fertility country. We analyse how attitudes, subjective norms and perceived behavioural control relate to the intention to have a child among childless individuals and one-child parents. Principal axis factor analysis confirms which items proposed by the Generation and Gender Survey (GGS 2005) act as valid and reliable measures of the suggested theoretical socio-psychological factors. Four parity-specific logistic regression models are applied to evaluate the relationship between the socio-psychological factors and childbearing intentions. Social pressure emerges as the most important aspect in fertility decision-making among childless individuals and one-child parents, and positive attitudes towards childbearing are a strong component in planning for a child. This paper also underlines the importance of the region-specific factors when studying childbearing intentions: planning for the second child significantly differs among the development regions, representing the cultural and socio-economic divisions of the Romanian territory.

## Introduction

In the 1990s, the period total fertility rate (TFR) dropped below the replacement level in many European countries. In some Central and Eastern European countries (CEE), which formed the ex-Soviet bloc, the TFR fell below 1.3, a phenomenon known as the ‘lowest-low fertility’ (Goldstein et al. 2009). The path to the lowest-low and low fertility differs across countries and it is largely explained by parenthood postponement (Billari et al. 2006; Sobotka 2004). However, while some demographers argue that the fertility decline in CEE is partly a response to economic uncertainty and social change in these countries (Frejka and Gietel-Basten 2016; Macura 2000; Perelli-Harris 2005; Sobotka et al. 2011), others emphasise the ideational changes, which partially replaced the traditional values with modern values—characteristic of democratic societies—after the collapse of the totalitarian regimes. It is thought that these new values, which are interpreted in light of the individualisation and post-materialistic theories (Inglehart 1997; Inglehart and Welzel 2005), triggered, at least partially, the second demographic transition (Lesthaeghe and Surkyn 2002; Philipov et al. 2006), which is responsible for the low fertility rates.

As one of the CEE countries, Romania experienced its lowest low fertility rate of 1.27 in 2002 (World Bank 2017) and has maintained relatively low levels of period fertility ever since. The fertility levels are low yet this is despite the traditional context of family formation encouraging the reproductive behaviour. Firstly, Romanians highly value being married and having their own family, perceiving childbearing as both a moral duty and a means of personal fulfilment (Rotariu 2006). Secondly, the mean ages at marriage and childbirth are low (though on the increase) compared to other European countries. This paradox, that emerged after the collapse of the Communist regime, is similar to that of Ukraine, Russia, Bulgaria, and Hungary, which have received far more attention in terms of explaining the country-specific factors associated with fertility behaviour (Billari et al. 2009; Perelli-Harris 2005, 2006; Philipov et al. 2006). Although the family formation paradox is similar, Romania departs from the other CEE countries by displaying the slowest development towards modernity and post modernisation, thus placing it among the most culturally traditional societies in Eastern Europe (Voicu 2008, p. 299). Therefore, understanding how Romanians decide to have children and what are the most relevant predictors of first- and second-order parity intentions complements the existing research on the former socialist countries.

In this paper, the study of fertility decision-making in Romania is based on the Theory of Planned Behaviour (TPB; Fishbein and Ajzen 2010). This paper considers the attitudes, the perceived social influence and the perceived control towards the intention to have a first child (among childless respondents) and a second child (among one-child parents). We refer to these two fertility intentions as parity-progression intentions in the rest of the article. The data are retrieved from the 2005 wave of the Generation and Gender Survey (GGS), which offers a unique opportunity to examine fertility intentions and its determinants based on items formulated according to the TPB (Vikat et al. 2007). The main aim of this paper is to understand if and how the attitudes, norms and perceived behavioural control are associated with the parity-progression intentions in Romania. We also investigate if and how the socio-economic and demographic characteristics are related to fertility intentions. We pay special attention to whether these characteristics change the relationship between the TPB psychological factors and intentions.

Romania has not previously been selected as a case study. Although Klobas and Ajzen (2015) included Romania in a comparative study on fertility determinants among several European countries using the TPB, they limited their focus on few demographic covariates such as age, education and partnership status. Moreover, the authors overlooked the regional demographic variability which is an important aspect of the regional development strategies at the European level. In this paper, we extend the previous research on fertility intentions by taking into account the regional differences within the country and a larger set of socio-demographic covariates. Romania is characterised by eight development regions established in accordance with the second-level regulations of the territorial classification (NUTS-2), a standard division at the European level. These regions differ in terms of cultural, economic and social factors, and these differences are reflected in the demographic behaviour as well (Sandu 1999, 2011a). This leads us to ask whether the regional breakdown of Romania is relevant for explaining fertility intentions. To the best of our knowledge, this study is the first empirical research to present an extensive and exclusive analysis of the Romanian fertility decision-making process of individuals aged 18–45 years, employing the TPB and taking the regional differences into consideration.

The remainder of the article is structured as follows. ‘The context of low fertility in Romania’ section presents the context of childbearing in Romania. ‘The TPB and Fertility Intentions’ section offers an overview of the TPB and discusses the latest research which applied it. The ‘Research questions**’** section illustrates the research questions in our study queries. ‘Data and methods’ section describes the data, the variables and the methods used. ‘Results’ section presents the results of the models. The ‘Discussion and conclusions’ section summarises and discusses the most relevant results.

## The context of low fertility in Romania

Following the transition from a communist to a democratic political regime in 1990, Romania entered a continuous process of declining fertility rates and increasing life expectancy (Bodogai and Cutler 2013; Ghețău 2008; Mureșan 2012). The most fertile age groups show a sharp postponement of first and second births, with weak signs of fertility recovery at later ages (Mureșan et al. 2008). Similar to other former socialist countries, Romania shows a pattern of early childbearing. However, the mean maternal age at birth has increased, from 23 years of age in 1990–1994 to 25.2 in 2000–2005 (Mureșan 2012, pp. 157–158). Nonetheless, compared with other European countries, the age of Romanian mothers at first birth is still low.

The decline in fertility in Romania after 1990 is correlated with women’s higher investment in tertiary education, work activity (Hoem et al. 2013; Mureșan and Hoem 2010) and increasing access to and use of modern means of birth control (Rotariu 2006). Mureșan (Mureșan and Hoem 2010; Mureșan 2007) underlines the negative educational gradient for the first, second and third births order. However, among highly educated women, those who are more family-oriented have their second birth sooner, thus hiding the true extent of the negative effect of a higher education on second births (Mureșan 2007). The negative educational gradient of childbearing is visible in both marriage and cohabitation, even if non-marital births are not as frequent in Romania as in other Western or Central European countries. Hărăguș (2010) used the Romanian GGS and underlined the strong negative association between educational attainment and first birth in cohabitation, this association being much more visible than for first marital births. In another study, Hărăguș (2008) showed that among cohabiting women, those with the highest education and socio-economic status living in urban areas tend to remain childless. Overall, since maternity leave is lower than an average wage in Romania, and since childbearing may cause mothers’ postponement of a career, one child might be a less costly family size for the most educated group of women, irrespective of the living arrangement.

Romania is also characterised by a limited availability of formal childcare and services (such as day-care centres, mother and baby units and foster homes) which should support families in need. After the political events in 1990, public childcare almost disappeared. Caregiver jobs, such as nannies or babysitting personnel, are rare and their financial costs are barely affordable, especially for families with a low socio-economic status and/or lone parents for whom the risk of poverty is high (Marin and Șerban 2008). Due to this, much of the care for the children is entrusted to mothers or to informal networks, namely grandparents (Castiglioni et al. 2016; Ministry of Labour, Social Solidarity and Family 2006). The unsatisfying childcare services and the lack of adequate financial resources (due to low salaries or unstable job market) to compensate the cost of a child challenge the equilibrium between work and family duties, especially for women (Mureșan and Hoem 2010; Popescu 2009; Vlăsceanu 2007). This, in turn, is a further reason for a decline in fertility (Bîrciu et al. 2009; Mureșan et al. 2008; Popescu 2009).

Additionally, the rigidity of the Romanian housing market, that restricts the access to homeownership due to high house prices, is among the structural factors which influence the family and fertility behaviour (Mureșan et al. 2008; Sobotka 2013). It is also worth noting that, in Romania, the family policy issues have received little attention, as the State has been reluctant to enact any family policy, especially due to the previously enforced pro-natalist measures.^1^

Based on value change studies, Romania is part of the ex-Soviet bloc of countries with a low post-materialistic index (Inglehart 1997; Inglehart and Welzel 2005; Sobotka 2008; Voicu 2008). Despite its very low fertility rates, Romania is a traditional society where family values remain important, and where family offers the greatest satisfaction to individuals. Marriage is seen as a trustful institution, and attitudes towards parenthood remain positive, with a high proportion of childless women who want at least one child (Rotariu 2006; Popescu 2009). Mureşan (Mureșan 2007; Mureșan 2012) explained that conservative values co-exist with a small percentage of the post-modern values seen among young, urban, working, higher-educated adults, whose attitudes towards childbearing converge on the intention to have only one child. This explanation suggests that the second demographic transition (SDT) dimensions could have slowly progressed in Romania, manifesting at an early stage.

Romania is characterised by large territorial differences. Namely, in 1998, eight development regions were identified,^2^ formed by a group of counties in the territorial contiguity. These regions are very different as far as GDP per capita, education, life expectancy and values are concerned. For example, the values and lifestyle of the North-East region differ from those of the other development regions such as the Centre, West and North-West regions. The mean age of mothers at first birth is lowest in the North-East and South-West regions (Mureșan et al. 2008). Several explanations for the regional differences have been posited. The first explanation is historical (Sandu 2011b): the domination of different empires in the Romanian history left cultural differences within the country (driven by influences of the Ottoman Empire for the South regions; the Austro-Hungarian Empire for the North-West, West and Centre; and the Russian Empire for the North-East and part of the South-East).^3^ The second explanation is economic and relates to the collapse of the Soviet industry, which triggered high dismissals of the workforce that were unable to reintegrate into any other fields of employment. The unemployment rate increased from 3.4% in 1990 to 7.1% in 2005 (International Monetary Fund 2015). However, while the decreasing trend in the number of employees has continued in most of the regions, this number has increased in the capital region, Bucharest-Ilfov. This area has also benefited in the highest degree from national and foreign investments (Lefter and Constantin 2009). Consequently, the Bucharest-Ilfov area is the most affluent, where the lifestyle is penetrated by more modern values. Sociologists and anthropologists studying space have put forth theories that spaces are not containers for society, or things by themselves, but are complex social constructions based on values and meanings individuals ascribe. Social relations and social identities are produced in a space where are becoming inscribed. In turn, a space reproduces and returns social relations and identities (Lefebvre 1991; Karlsson 2003; Kearney 2004). Therefore, the eight Romanian development regions are seen as socio-cultural matrices, each one standing for a mental pattern with physical boundaries and well-configured social identities, which influence individual expectations and behaviours (Sandu 2011a).

## The TPB and fertility intentions

### Theoretical considerations

The TPB is a socio-psychological model that allows the studying of decision-making processes which account for intentional behaviours. The theory was formulated by Ajzen and Fishbein (1974) and developed by Ajzen (1991, 2005, 2011), who included and operationalised the perceived behavioural control component. Fishbein and Ajzen (2010) consider attitudes, subjective norms and perceived behavioural control as best predictors of any behavioural intention. The model has been tested and validated in numerous studies on various decision-making processes in different contexts, ranging from condom use (Ajzen et al. 1996; Albarracin et al. 2001), health and wellbeing (Conner et al. 2002) to workplace (Greaves et al. 2013) and digital piracy (Yoon 2010). Since the announcement of the TPB, an increasing number of demographers, socio psychologists and other scholars interested in the fertility behaviour domain have applied the theory in part or in its entirety to better understand reproductive decision-making at the micro-level (Billari et al. 2009; Dommermuth et al. 2011; Ajzen and Klobas 2013; Jaccard and Davidson 1975; Miller and Pasta 1995; Schoen and Tufis 2003).

A behavioural intention is defined as a plan or a likelihood that the individual will act in a particular way, in a specific situation, in a given context and at a given time framework. It is the proximate antecedent of a certain behaviour that can either happen or not. As Fishbein and Ajzen (2010, p. 40) underlined, ‘the term intention (…) refers to the subjective probability of performing a behaviour’. The three determinants of intentions (i.e. attitudes, subjective norms and perceived behavioural control) are considered evaluations towards performing the behaviour that are formed through cognitive and emotive processes. These evaluations are influenced by the different beliefs people hold.

A schematic presentation of the TPB (Appendix, Scheme A1) contributes to the understanding of how behavioural, normative and control beliefs influence attitudes, subjective norms and perceived behavioural control, which, in turn, influence intention. The intention is ultimately the proximate antecedent of the actual behaviour.

*Attitudes* to a behaviour represents people’s internal evaluations that performing a behaviour will have positive or negative outcomes for them. In general, the more positive is one’s outcome of performing the behaviour, the more favourable one’s attitude is towards the behaviour.

*A subjective norm* is a person’s perception of the psychological support or pressure that significant others exert for performing the behaviour. It is called ‘subjective’ because, on the one hand, it relates to the singular perception of the individual, but on the other hand, the perceived norms might not accurately match the actual opinions of other people (or the wider societal norms). In general, as more important referents approve than disapprove of a specific behaviour, and as more of them actually perform that behaviour, the more likely individuals are to perceive a greater social pressure towards performing the behaviour.

*Perceived behavioural control* reflects people’s perceptions of being able or not to perform the behaviour. This concept is similar to Bandura’s (1977) self-efficacy concept in that it articulates the people’s perceptions of the ease or difficulty to perform the behaviour. A good example to understand better the perceived control component of the TPB is with income: the wealthy might believe that they cannot afford to have a child, while those less wealthy might think they are financially independent enough to have a child. Therefore, financial status is not the issue: what matters is the *conviction* of having the financial resources to raise a child. Since it is a perception, it may not reflect reality, just as the case with subjective norms.

Some variables often studied in demographic research (such as income, education, religion and parity) are treated as ‘*external’ variables* in social and psychological studies of fertility intentions; these are considered external to the cognitive structure associated with making a specific decision (Ajzen 2005; Billari et al. 2009; Dommermuth et al. 2011). The TPB distinguishes between two types of external variables: actual behavioural control and background factors.

*Actual behavioural control* refers not only to the person’s skills and abilities necessary to perform the behaviour but also to different factors that may enable (*enablers*) or disable (*constraints*) the individual to act as intended. Under ideal conditions of measurement and operationalisation of the factors (Ajzen 2005, p.134), the effect of actual enablers and constraints on intentions is mediated by the perceived behavioural control.

*The background factors* are clustered into individual, social and informational categories. As stated above, under ideal circumstances, the background factors influence the beliefs people hold and, in turn, influence the theory’s proximal determinants.

Many empirical applications of the above theoretical framework used simplified versions of the original formulation of the TPB model. Ajzen (2005, pp. 135–136; 2011) acknowledged several studies which considered a set of background factors as direct influences on the intention and behaviour of interest. Several studies of fertility intentions also used a simplified approach by proposing a direct association between the background factors usually represented by socio-demographic variables and parity decisions. For example, Billari et al. (2009) estimated not only the intermediated effect of the background factors on the intentions through attitudes, subjective norms and perceived behavioural control but also the direct effect of these factors on fertility intentions. Proving the direct effects of the demographic and socio-economic characteristics of individuals on fertility intentions, Billari et al. (2009) argued: ‘If the TPB is “*true*” under ideal conditions of measurement and operationalisation of the components, the direct effect of background factors should be absent’ (p. 447). Dommermuth et al. (2011) took a similar approach and estimated the direct effect of both the objective socio-economic (the actual behavioural control) and the demographic characteristics of individuals (background factors) on the timing of the intention to have the first or second child in Norway.

### Key research in the field

Several scholars who used the TPB have underlined the importance of studying the determinants of reproductive intention within the fertility decision-making context. For example, Klobas and Ajzen (2015), who examined between-country differences in the effects of attitudes, subjective norms and perceived behavioural control on fertility intention, demonstrated the importance of using the TPB model for understanding fertility intentions. They claimed that socio-psychological factors explain the decision to have a child much better than national contextual differences alone or in combination with individual differences (Klobas and Ajzen 2015).

Using graphical models to study the Italians’ fertility intention and their outcomes based on the TPB, Mencarini et al. (2015) found that fertility realisation does not depend on attitudes, perceived behavioural control and subjective norms. The TPB factors were associated instead with fertility intentions, as the theory posits.

Dommermuth et al. (2011) investigated the role of attitudes, subjective norms and perceived behavioural control on the time frame in which individuals intended to have a child (‘now’ and ‘within the next three years’). They found that subjective norms had a significant effect on the timing of the intentions of childless people and one-child parents to have a child. The more childless individuals and first-parity parents felt that their intentions to have a child were supported by their families and friends, the more likely they were to want a child in the short term (‘now’) compared to later (‘within the next three years’). Perceived behavioural control was a significant determinant for both groups: people who considered themselves better able to cope with having a child were more likely to intend to have a child in the short term (‘now’) rather than later (‘within the next three years’). However, this effect disappeared when the authors controlled for demographic background variables. It seems that for the Norwegian case, the effect of perceived control on the timing of having a child varies considerably with personal circumstances.

A study on fertility intentions in Bulgaria revealed that perceived behavioural control had an effect on the decision to have a second child and subjective norms were the most influential on the intentions to become a parent (Billari et al. 2009).

Even though some of these studies evoked the context-specific influences on the fertility decision-making process, none considered the regional differences in forming fertility intentions.

## Research questions

We apply a simplified version of the TPB model in order to estimate the effects of attitudes, subjective norms and perceived behavioural control factors on the intention to have a first or second child in Romania, following the approach proposed by Dommermuth et al. (2011). The authors consider a direct relationship between the actual behavioural control and background factors on fertility intentions. Differently from Dommermuth et al. (2011), we include the development regions as proxy for the cultural and socio-economic variation across the country.

**Scheme 1:**
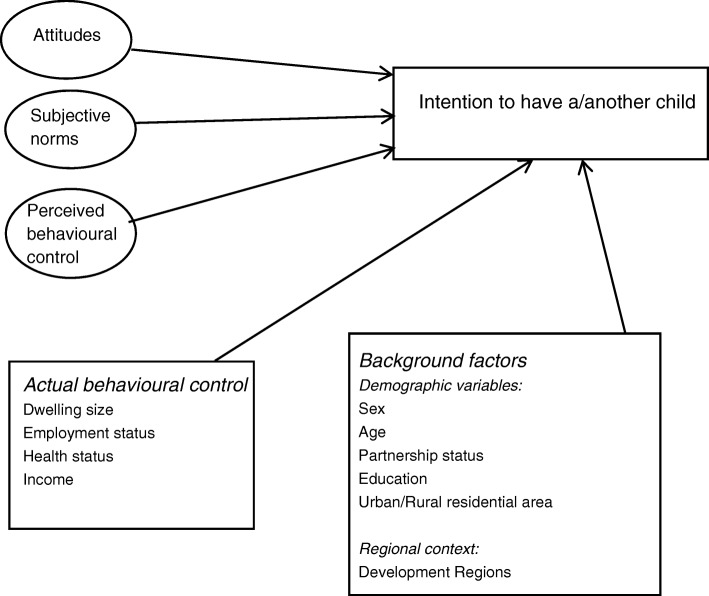
Simplified model of fertility decision-making based on TPB

Scheme 1 is an image of the simplified model proposed in this paper.

Adopting the above simplified version of the theoretical model, the following research questions guide the analysis:

1. Do attitudes, subjective norms and perceived behavioural control explain simultaneously the intention to have a first or second child?
2. How are the objective socio-economic and health conditions of the childless individuals and one-child parents associated with fertility intentions?
3. Do the above relationships hold once the demographic background factors are controlled for?
4. Are the Romanian development regions relevant for explaining fertility intentions?

## Data and methods

### Database and sample

We use the 2005 wave of the Romanian GGS to answer to the research questions. The GGS takes a life course approach to the most important individual decisions, such as leaving home, partnership formation, marriage, childbearing, retirement, work-family balance, gender relations and intergenerational exchanges. Besides the fertility theme that it captures, the database is chosen because it contains questions on fertility intention formulated using the TPB. In accordance with the theory, the intention and its determinants are measured on the same level of specificity, namely on a time framework of planning to have a child within the next three years. Hence, it meets the principle of compatibility criteria that Ajzen (2005) and Fishbein and Ajzen (2010) warn about.

The 2005 wave of the GGS surveyed 11,986 cases. Of interest to this research are two samples: one consists of 1683 childless men and women, among which 1081 are men and 602 are women, and the second one consists of 1521 one-child parents, among which 735 are men and 786 are women. The analytical samples emerged after applying the necessary filters to obtain individuals who do not have any children (for the childless individuals group), who have only one child (for the one-child parents group), in any type of union (married, cohabiting or living apart together [LAT]) and all aged 18–45 years.^4^ Women who declared that they were already pregnant at the time of the interview were excluded from the analysis for both groups.

### Methods and model specification

The 2005 GGS provides measurements of attitudes, perceived norms and perceived behavioural control towards having a child derived from the TPB. Factor analysis (using the principal axis factoring algorithm [PAF]) is performed to identify the items that load high on the TPB theoretical components for the two subsamples. The latent dimensions that underline the attitudes, normative and perception beliefs towards having the first and second child are thus identified.

The two dependent variables, the intention to have a first child (among the childless individuals) and the intention to have a second child (among the one-child parents), are estimated using a standard logistic model:

1 $$ P\left(\mathrm{Intention}\right)\kern0.5em =\kern0.5em \frac{\exp \left(\eta \right)}{1+\exp \left(\eta \right)} $$

where *η* is a function of the three main latent variables, identified by the exploratory factor analysis, and of the socio-economic and demographic covariates.

Four logistic regression models are run using parity-specific contexts. The first logistic regression model contains only the socio-psychological variables, represented by the attitudes, subjective norms and perceived behavioural control factors. These variables are measured as factor scores estimated by the factor analysis (see ‘Attitudes, subjective norms and perceived behavioural control’ section).

In the second model, we add the set of the ‘actual behavioural control’ variables measured through the health conditions and socio-economic situation of the respondents (income, health status, employment status and dwelling size).

In the third model, standard demographic variables, represented by measures of partnership status, education, age, sex and residential area (urban/rural), are added as demographic background factors of control.

The fourth regression model is the most complex since it contains the latent TBP variables, the actual behavioural control, the demographic background variables and the development regions. The eight Romanian development regions are the last background variable added in this fourth model, controlling for the regional context.

Some variables, namely income and, to a lesser extent, fertility intentions, have a relevant number of missing values which have been corrected using a generalisation of the ‘hot-deck’ imputation method^5^ to save as much information as possible from the samples of interest.

All the statistical data analyses were performed using R language (R Development Core Team 2016; Field et al. 2012).

### Dependent variables: intention to have a first or second child

The dependent variables used in this paper are *the intention to have a first child within the next three years and the intention to have a second child within the same time period*. The variable ‘Do you intend to have a/another child during the next three years?’, with four response categories (‘definitely yes’, ‘probably yes’, ‘probably no’, ‘definitely no’), is transformed into a dummy variable with the reference category ‘not wanting a child during the next three years’ (‘probably no’ and ‘definitely no’ take the value of zero). The same transformation is made for the one-child parents group.

As noted earlier, the birth intention variables have a limited number of missing values. After imputation, the number of item non-responses for the first-child intention fell from 33 to just 1, and the one for the second child intention fell from 72 to none.^6^

### Attitudes, subjective norms and perceived behavioural control

In the GGS, three blocks of questions are used to operationalise attitudes, subjective norms and perceived behavioural control (Vikat et al. 2007). The attitudes towards having a child are measured as respondents’ answers to eleven items using a five-point Likert response scale, where 1 means ‘much better’ and 5 means ‘much worse’. Respondents are asked to evaluate the anticipated effect on having a child on different outcomes such as ‘your financial situation’ and ‘your sexual life’ (see Table A.3 in the Appendix for a detailed list of the items). Each of these items is introduced by the question: ‘Suppose you will have a/another child within the next three years. On a scale from 1 to 5, where 1 means “much better” and 5 means “much worse”, would it be better or worse on...?’

Since we want to identify two distinct latent factors called ‘Benefits’ and ‘Costs’, the items associated with the Benefits factor have been reversed to ease the interpretation of its possible positive effects on the intention to have a first child.

Subjective norms are measured through three items asking the participants to rate the extent to which they agree that three groups of normative referents—parents, relatives and friends—hold about them having the first or second child. These items are measured on a five-point response scale, ranging from 1 (‘strongly agree’) to 5 (‘strongly disagree’). These response scales have been reversed so that the higher scores represent higher perceived social pressure; as such, a positive effect corresponds to a positive coefficient in the logistic regression models. All three items were introduced by the following question: ‘Although you may feel that the decision of whether or not to have a/another child is yours, it is likely that others have opinions about what you should do. On a scale from 1 to 5, where 1 means “strongly agree” and 5 means “strongly disagree”, to what extent do you agree with these statements?’

The GGS provides nine items to measure the perceived behavioural control. Respondents are asked to what extent their intention to have a child depends on the following: financial situation, work, housing conditions, health, having a suitable partner and availability of childcare. The values on the response scale for these items (1 = ‘not at all’; 2 = ‘a little’; 3 = ‘quite a lot’; 4 = ‘a great deal’) have been reversed for easier interpretation as possible positive effects to overcome constraints on fertility intention in the regression analysis. As Klobas (2010) justified, perceived control of the intention to have a child may be interpreted in the respondent’s evaluation as it being possible to find a balance between work and childrearing duties, to provide space in the dwelling or move to a new house with enough space for the extended family, or that the respondent is able to financially support the child. The items are introduced by asking: ‘How much would the decision whether to have a/another child within the next three years depend on the following…?’

We ran an exploratory factor analysis on these three sets of items by using the PAF algorithm^7^ (Thurstone 1947; Gaskin 2016). Since the items are measured on Likert-type scales, the analysis was conducted on the matrix of polychoric inter-item correlations, which is a special case for latent variable modelling (Baglin 2014; Ekström 2011; Holgado-Tell et al. 2010; Norman 1979; Olsson 1979). We identified two factors—‘Benefits’ and ‘Costs’—for the Attitudes component, for both childless individuals and one-child parents. The ‘Benefits’ factor represents beliefs about the benefits of having a child, while the ‘Costs’ factor represents beliefs about the financial or personal losses associated with having a child. We also identified one factor for the perceived behavioural control (PBC) and one for the subjective norms (SN).

Tables 1 and 2 present on overview of the items that capture the factors proposed by the TPB for the childless individuals and one-child parents.

**Table 1:** Factor loadings of items for attitudes, subjective norms and perceived behavioural control for childless respondents

	Factor 1 Positive attitudes: benefits	Factor 2 Negative attitudes: costs	Factor 3 Perceived behavioural control	Factor 4 Subjective norms
“Suppose you will have a/another child during the next 3 years, would it be worse or better for...?”				
The possibility to do what you want	0.02	0.91		
Your employment opportunities	0.01	0.75		
Your financial situation	− 0.12	0.63		
What people around you think of you	0.61	− 0.13		
Joy and satisfaction you get from life	0.78	− 0.09		
The closeness between you and your partner/spouse	0.71	− 0.04		
The care and security you may get in old age	0.79	0.12		
Certainty in life	0.82	0.06		
The closeness between you and your parents	0.65	− 0.05		
“How much would the decision on whether to have a/another child during the next 3 years depend on the following?”				
Your financial situation			0.79	
Your work			0.77	
Your housing conditions			0.77	
Your health			0.75	
You having a suitable partner			0.70	
Your partner’s/spouse’s work			0.74	
Your partner’s/spouse’s health			0.80	
Availability of childcare			0.64	
“Others might think about you having a/another child during the next 3 years, do you disagree or agree with these statements?”				
Most of your friends think that you should have a/another child				0.89
Your parents think that you should have a/another child				0.91
Most of your relatives think that you should have a/another child				0.96
Cronbach alpha	0.84	0.75	0.89	0.94
KMO		0.83	0.87	0.76
RMSR	0.05		0.08	0

Items with communalities less than 0.4 and with factor loadings over 0.5 were retained in the model; RMSR: the root mean square of the residuals; a value less than 0.08 is generally considered a good fit (Hu and Bentler 1999); KMO: Kaiser-Meyer-Olkin measure of sampling adequacy; values higher than 0.7 are generally considered good, suggesting sample size and data are appropriate for factor analysis

Source: GGS, Romania 2005, own computations

**Table 2:** Factor loadings of items for attitudes, subjective norms and perceived behavioural control for one-child respondents

	Factor 1 Positive attitudes: benefits	Factor 2 Negative attitudes: costs	Factor 3 Perceived behavioural control	Factor 4 Subjective norms
“Suppose you will have a/another child during the next 3 years, would it be worse or better for...?”				
The possibility to do what you want	0.02	0.92		
Your employment opportunities	− 0.01	0.77		
Your financial situation	− 0.16	0.53		
What people around you think of you	0.50	− 0.19		
Joy and satisfaction you get from life	0.74	− 0.09		
The closeness between you and your partner/spouse	0.72	− 0.04		
The care and security you may get in old age	0.78	0.11		
Certainty in life	0.83	0.02		
The closeness between you and your parents	0.73	0.00		
“How much would the decision on whether to have a/another child during the next 3 years depend on the following?”				
Your financial situation			0.73	
Your work			0.70	
Your housing conditions			0.71	
Your health			0.81	
You having a suitable partner			0.69	
Your partner’s/spouse’s work			0.72	
Your partner’s/spouse’s health			0.81	
Availability of childcare			0.66	
“Others might think about you having a/another child during the next 3 years, do you disagree or agree with these statements?”				
Most of your friends think that you should have a/another child				0.91
Your parents think that you should have a/another child				0.90
Most of your relatives think that you should have a/another child				0.99
Cronbach alpha	0.83	0.74	0.87	0.92
KMO		0.83	0.86	0,74
RMSR	0.04		0.1	0

Items with communalities less than 0.4 and with factor loadings over 0.5 were retained in the model; RMSR: the root mean square of the residuals; a value less than 0.08 is generally considered a good fit (Hu and Bentler 1999); KMO: Kaiser-Meyer-Olkin measure of sampling adequacy; values higher than 0.7 are generally considered good, suggesting sample size and data are appropriate for factor analysis. Source: GGS, Romania, 2005, own computations

### Actual behavioural control

As mentioned before (‘Theoretical considerations’ section), the actual behavioural control (or the objective measures of control) refer not only to the person’s skills and abilities to perform the behaviour but also to different factors (enablers and constraints) that may enable or disable the individual to act as intended.

Following Dommermuth et al. (2011), and according to the data collected in the frame of the GGS project, we included individuals’ socio-economic characteristics such as dwelling size, employment status, health status and income. Dwelling size is an indicator of the housing condition and it is measured by the number of rooms at the respondent’s dwelling. Employment status has been transformed from a categorical variable into a dummy variable, with the reference category ‘unemployed’. The health status variable has been also recoded as a dummy variable, with the reference category ‘bad health’.

Income is self-reported and measured in the national currency, RON. The item non-response for this variable is high for both samples: for the childless individuals group, the item non-response is 26% and for the one-child parents group it is 16%. Moreover, these missing cases can hardly be assumed to be at random because respondents with either a high or very low income are less likely to report their incomes (Soley-Bori 2013), suggesting that the probability of the missing values depends on some unobserved variables. Thus, a previous treatment of the data to reduce this lack of information has been performed, applying the already mentioned ‘hot-deck’ imputation method (see ‘Methods and model specification’ section). After correction, the percentage of non-responses for income fell to around 10% for both groups.

### Background factors

The covariates included in this group are age, gender, union status, education residential area and the development regions. Age is categorised in groups (18–29, 30–35 and 36–45 years old). Union status includes the following categories: no partner, LAT, married and cohabiting. The respondents’ levels of education are recoded into two categories: secondary (comprising individuals with a maximum of secondary level of education) and tertiary education (first and second stages of tertiary education). The primary and secondary levels are merged because of the small number of individuals in this category. The residential area is distinguished between rural and urban, with the former used as the reference category.

The development regions are recoded in the logistic regression as seven dummy variables, with one reference category (the Bucharest-Ilfov region).

## Results

### Descriptive results

Table 3 shows the statistics of the covariates by parity context. Because the TPB factors, the dwelling size and income are quantitative variables, their values are presented as means (standard deviations between parentheses). The percentage of childless respondents intending to have a child (50.4%) is almost equal to that of those not intending to have a child (49.4%), while the majority of the one-child parents plan to bear another child (68.3%). The childless respondents are younger, more often single, unemployed and have a lower income than those with one child. In both subsamples, most respondents have a tertiary education level (84.6% and 85.3%), are employed (63.1% and 78.3%) and in good health (63.1% and 78.3%). The majority of both subsamples live in urban areas (65.1% and 65.9%) and are quite homogenously distributed among the eight development regions.

**Table 3:** Means, standard errors and percentages of descriptive statistics by parity context of the variables used in the study

Variables in the model	Childless	One-child parents
Fertility intention (%)		
Yes	50.4	31.6
No	49.5	68.3
TPB factors (mean scores (s.e.))		
Positive attitudes: benefits	0.06 (0.03)	0.03 (0.03)
Negative attitudes: costs	− 0.10 (0.03)	− 0.03 (0.03)
Perceived behavioural control	− 0.02 (0.03)	0.03 (0.03)
Subjective Norms	− 0.01 (0.03)	0.01 (0.03)
Dwelling size (mean n. of rooms (s.e.))	2.59 (0.99)	2.61 (0.03)
Employment status (%)		
Not employed	36.9	21.7
Employed	63.1	78.3
Health status (%)		
Bad health	7.7	11.3
Good health	92.3	88.7
Income (in RON - mean (s.e.))	235.62 (11.83)	306.88 (16.77)
Gender (%)		
Female	64.2	48.3
Male	35.8	51.7
Partnership status (%)		
No partner	58.4	6.7
LAT	17.1	1.5
Married	20.1	87.1
Cohabiting	4.5	4.7
Education (%)		
Tertiary	84.6	85.3
Secondary or less	15.4	14.7
Residential area (%)		
Rural	34.9	34.1
Urban	65.1	65.9
Age class (%)		
18–29	63.5	18.3
30–35	18.9	30.6
36–45	17.7	51.1
Developmental regions (%)		
Bucharest-Ilfov	12.6	11.1
Centre	12.8	10.8
North-West	13.0	12.2
West	13.4	10.3
South-West Oltenia	9.2	12.4
South-Muntenia	13.3	16.5
Total (N)	1683	1521

Where the sum of the percentages is not equal to 100, the reason may be one or more of the following: missing values excluded, refusals, “do not know”, a rounding effect

### Multivariate results

Table 4 presents the results from the regression models. As the intention to have the first child is qualitatively different from the decision to have a second one, we ran parity-specific models and conducted step-wise analyses. The results are presented as odds ratios.

**Table 4:** Effects of factors from the theory of planned behaviour, objective measures of control and background demographic variables for childless individuals and one-child parents

	Childless				One-child parents			
	Model 1	Model 2	Model 3	Model 4	Model 1	Model 2	Model 3	Model 4
Factors for the Theory of Planned Behaviour								
Positive attitudes: benefits	1.60***	1.64***	1.61***	1.61***	2.40***	2.31***	2.27***	2.33***
Negative attitudes: costs	0.69***	0.69***	0.62***	0.61***	0.50***	0.49***	0.51***	0.48***
Perceived behavioural control	1.22*	1.10	1.01	0.99	1.14	1.13	1.21	1.17
Subjective norms	3.22***	2.81***	2.48***	2.47***	3.03***	3.11***	2.81***	2.81***
Actual behavioural control								
Dwelling size		0.79**	0.89	0.87		0.92	0.92	0.95
Employment status (ref. Not employed)								
Employed		3.28***	2.69***	2.68***		0.73	1.07	1.06
Health status (ref. bad health)								
Good health		0.93	0.99	0.99		2.18*	1.57	1.80
Income		1.00	0.99	0.99		0.99	0.99	0.99
Demographic background factors								
Gender (ref. male)								
Female			1.66*	1.63*			0.69	0.69
Partnership status (ref. No partner)								
LAT-Living apart together			1.63*	1.61*			0.35	0.29
Married			3.63***	3.73***			0.85	0.72
Cohabiting			4.10**	4.25**			1.22	1.40
Education (ref. tertiary)								
Secondary or less			1.45	1.45			0.94	0.86
Residential area (ref. rural)								
Urban			1.21	1.26			0.79	0.87
Age class (ref. 36–45)								
18–29			1.68*	1.64			7.34***	7.57***
30–35			2.86**	2.75**			3.73***	3.75***
Development regions								
Regions (ref. Bucharest-Ilfov)								
Centre				1.23				1.53
North-West				1.07				2.15
West				0.76				0.72
South-West Oltenia				1.21				1.75
South-Muntenia				1.29				0.89
South-East				0.82				1.57
North-East				1.05				2.54*
*N*	1023	921	921	921	1039	948	948	948
-2LogLikelihood	− 509.9413	− 435.7271	− 403.0342	−401.1143	− 430.1782	− 383.6826	− 347.008	− 338.4125
AIC	1029.9	889.45	841.88	850.23	870.36	785.37	728.02	724.83

*AIC* Akaike Information Criteria

Source: GGS, Romania, 2005, own computations

*p < 0.05; **p < 0.01; ***p < 0.001

To answer the research question concerning whether attitudes, subjective norms and perceived behavioural control can explain simultaneously the intention to have the first or second child, we consider particularly the first regression model.

We notice that in model 1, the TPB factors explain the childbearing intention only among the childless respondents. Once we introduce the covariates for the dwelling size, employment status, health and income, the association between the perceived control and the first-child intention loses its significance. The perceived behavioural control remains non-significant also in the third model (without regions) and fourth model (which includes regions). Among one-child parents, the perceived behavioural control is not significant in any of the four regression models.

Among the TPB socio-psychological variables, the normative influences have the strongest effect towards the childbearing intentions, for both groups. On one side of the spectrum, the benefits a child is thought to bring to the respondents’ lives are positively associated with parity-progression intentions. On the other side of the spectrum, the costs associated with having a child decrease the likelihood of planning another child within the next 3 years. This association stays significant in all four logistic regression models, even when objective measures of control, and all the background factors are added, suggesting a powerful relationship between the negative beliefs towards childbearing and the decision not to plan a child.

Model 2 addresses our second research question, that is ‘How are the objective socio-economic and health conditions of the childless individuals and one-child parents associated with fertility intentions?’ While employment status has a significant effect on the intention to become a parent, it has no effect on the second-order parity intention. A counter-intuitive finding is that the bigger the dwelling size (measured in number of rooms), the lower the likelihood of intending to become a parent. Dwelling size does not play a significant role in the second-order parity decision.

Among one-child parents, a better health is associated with the intention to plan the second child, while it has no significance for the childless respondents. This result might be because good health acts as a determinant to plan another child; thus, those who report bad health or illnesses might want to wait until their health improves. Contrary to our expectation, there is no relationship between income and childbearing intentions; this counter-intuitive finding is discussed in ‘Discussion and conclusions’ section.

We answer the research question ‘Do the above relationships hold once the demographic background factors are controlled for?’ within the context of the third logistic regression model (model 3): once we control for the background variables, neither the effects of the main TPB components nor those of the employment status or income, as objective measures of control, significantly change. These results suggest that the demographic factors do not change the relationship between the socio-psychological variables and the parity-progression intentions. There remains a strong link between the TPB factors and fertility intentions, a finding consistent with previous research (Billari et al. 2009; Dommermuth et al. 2011). The only notable exceptions are the effects of the dwelling size for the childless individuals and of health for the one-child parents, which both disappear after introducing the demographic characteristics. The negative association between the dwelling size and the intention to have a first child is accounted by the partnership status. This suggests the importance of being in a relationship and perhaps the partners’ wish to plan the first child among the childless individuals’ fertility intention. Among one-child parents, age accounts for their health status, suggesting that the biological clock might be more important than health for those who intend to have a second child.

Among the demographic background variables, age is the only one showing significant effects on childbearing intentions for both childless individuals and one-child parents. The childless respondents aged 30–35 years have the highest likelihood of intending to have a child within the next 3 years compared with those aged 36–45. For one-child parents, the highest likelihood of planning the second child belongs to the respondents aged 18–29 years old. This finding might also be counter-intuitive, but among the one-child parents, a high percentage of the younger respondents (18–29 years old) expressed their intention to have a second child (see Appendix, Table A.2). Compared with childless men, childless women have a higher likelihood of wanting a child within the next 3 years, whereas gender is not associated with the second childbearing intention.

The partnership status is only significant for the childless adults. Being married is the most important partnership type in the association with planning the first child, followed by those cohabiting and those in a LAT relationship. Finally, no significant difference has been found in the intention of having a child between people living in urban and rural areas.

To explore whether the development regions are relevant for explaining fertility intentions, we introduce seven dummies for the development regions in model 4, with the region of Bucharest-Ilfov taken as the reference category. None of the development regions are associated with the intention to have a first child. However, compared with living in the Bucharest-Ilfov region, living in the North-East region increases the likelihood of planning a second child. The result may capture these two regions different cultural and socio-economic characteristics.

It is worth noting that the full model (model 4), including the socio-psychological variables, the actual behavioural control variables and all the background factors, is the preferable one, as it captures most of the data variability.^8^

## Discussion and conclusions

This paper enlists the Theory of Planned Behaviour to improve the understanding of how childless individuals and one-child parents form their childbearing intentions. We applied the TPB to the Romanian case, a former ‘lowest low fertility’ country, which had a rather constant low fertility rate for almost a decade (1.3 children per female between 1995 and 2005). Inspired by Billari et al. (2009), we applied a simplified version of the Fishbein and Ajzen (2010). We adopted Dommermuth et al. (2011) strategy by considering the actual behavioural control and the background variables as having a direct relationship with the fertility intentions.

Among the background factors, we paid special attention to the regional context in order to account for the respondents’ socio-economic and cultural interdependency. The values, the lifestyle and the economic power differ across the eight development regions in Romania, which can be considered as spatial matrices (Lefebvre 1991; Kearney 2004) that influence individual expectations and behaviours (Sandu 1999, 2011a, 2011b).

**Fig. 1:**
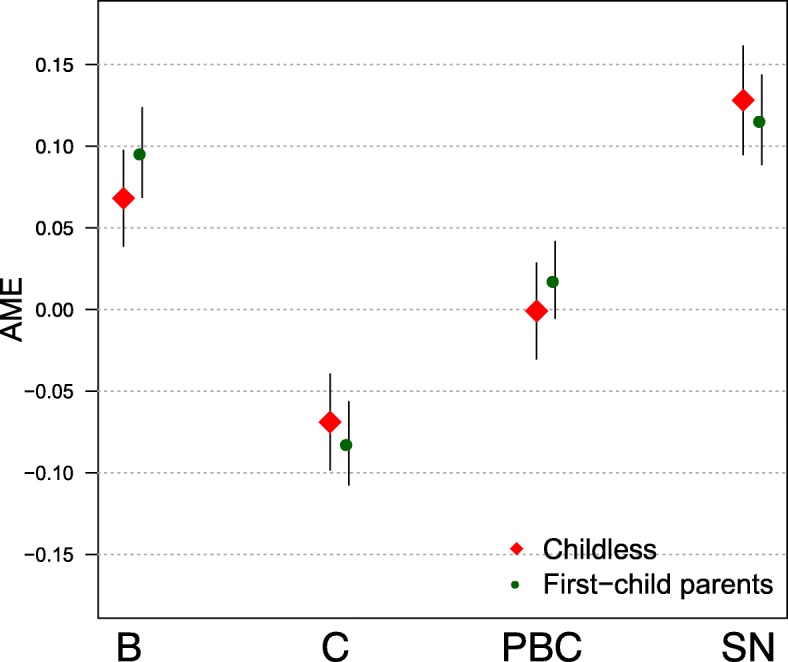
The average marginal effects (AME) of the socio-psychological factors from the Theory of Planned Behaviour on childbearing intentions from the full regression model. Notes: The full model includes the attitudinal factors benefits (B) and costs (C), the perceived behavioural control (PBC), and the subjective norms factors (SN); the actual behavioural control variables, the demographic background factors and dummies for the development regions; the model is run separately by parity-specific context (childless individuals and first-child parents). Black lines denote the 95% confidence interval. Data source: GGS, 2005, own computations

We used data from the 2005 wave of the GGS and performed four logistic regression models. Figure 1 summarises the main results, showing the average marginal effects of the socio-psychological variables on fertility decision-making from the full model.

Social pressure exerted through the subjective norms (SN) and the benefits a child is expected to bring (B) are highly associated with both childless individuals’ and one-child parents’ fertility intentions, net of other covariates. The negative expectations a child is thought to bring to individuals’ lives, represented by the expected costs (C), are negatively associated with childbearing intentions for both groups, while the perceived behavioural control (PBC) has little importance in the formulation of childbearing intentions.

These findings are in line with the traditional family values of the Romanian society, where societal norms discourage childlessness and push towards large(r) families (Popescu 2009; Rotariu 2006). Evidence for Bulgaria, another former socialist country, shows that normative pressure is more relevant for intentions to become a parent than for intentions to have a second child (Billari et al. 2009). There might be a pattern for the former socialist countries to ground the decision to become a parent within the societal family norms and pressure.

The positive attitudes towards having a child are also important in forming the parity-progression intentions for both groups of respondents. This is not necessarily the case for other countries, where a strong positive effect on the fertility intentions was observed only among one-child parents (for Norway: Dommermuth et al. 2011; for Bulgaria: Billari et al. 2009). This result can be explained by family values being more important within the Romanian traditional society than in other countries as Romanians consider having children as a major life fulfilment (Rotariu 2006). Among one-child parents, the decision to plan a second child might be driven by the perceived positive outcomes the first child has brought. In both samples, however, those who assess the child as a negative outcome to their lives perceive the child as a cost, and this reduces the intention of planning a child within the next 3 years, a finding in accordance to the TPB theory (Ajzen 2011).

The effect of perceived behavioural control, which captures the individuals’ perception that they are able to cope with having a first or a second child, is positive and significant only for the intention to have the first child. However, the effect of the perceived behavioural control disappears when the socio-economic characteristics of the childless respondents (the actual behavioural control variables) are controlled for: the housing size and being employed capture the effect of the perceived behavioural control factor. Employment status is a significant enabler of the intention to become a parent. However, it does not relate to the intention to have a second child. Dwelling size shows a counter-intuitive result: childless individuals living in a large house have a lower likelihood to intend to become a parent. This result might relate to the intergenerational co-residence, a common living arrangement in Romania (Castiglioni et al. 2016), which we did not account for. It could be that the larger dwelling size relates to respondents living with their parents or their partner’s parents, making them less likely to plan to enter parenthood until they obtain their own dwelling. Since dwelling size and being employed capture the effect of the perceived control childless individuals think they have over their life, the results indicate that this perception is overestimated compared with the reality, underlining the importance of the actual life conditions of these respondents.

Among the background variables, once the partnership status is considered, the significance of the dwelling size disappears. This suggests the importance of being in a relationship and perhaps the partners’ wish to plan the first child for the childless individuals’ fertility intention.

Income is not significantly associated with childbearing intentions once attitudes, employment and the background factors are controlled for. Thus, we can conclude that money is not a deciding factor for planning a child. Even if at first glance this finding is surprising, given the traditional context of the value system Romania still has compared with other European countries (a low post-materialistic index; family values offer the greatest satisfaction to individuals; marriage is a trustful institution; attitudes towards parenthood remain positive), it might come naturally for Romanians to care less for the material aspects when planning to have a child (Rotariu 2006; Voicu 2008). However, this result emphasises even more the fertility paradox of Romania where fertility rates are low despite a traditional setting of family formation where marriage and parenthood are seen as major life satisfactions. More research is needed to unpack the reasons why fertility rates are depressed, especially in a context where money does not seem to affect childbearing intentions.

The background variables are associated with the above relationships, which confirm the importance of considering them as control factors, as did Billari et al. (2009) and Dommermuth et al. (2011). Among them, the relevance of the regions in explaining fertility-decision making is limited but not negligible. Compared with the Bucharest-Ilfov region, living in the North-East region increases the likelihood of planning a second child. This result is explained by the fact that these two regions have different cultural and socio-demographic characteristics; for example, the average cultural modernity in Bucharest-Ilfov, the capital region, is the highest across country, whereas the North-East region belongs to the rather traditionalist value system, where individuals value families with more than one child. Demographic indicators underline that in the North-East, women have the highest parities and become mothers earliest compared with other regions. At the same time, Bucharest-Ilfov region has the lowest rates of transitions to a second birth (Mureșan et al. 2008). Having more or less children might be related to individuals’ social identity which is produced differently in these two specific regions via social interactions and shared meaning of what a ‘proper’ family size should be. Moreover, given that among the development regions in Romania, the North-East is the one with the highest poverty risk, it may be that intending to have a second child reflects the lack of opportunities to invest in longer education or careers and individuals are socialised or pressed to have larger families. Further research is needed to understand the reasons why people in North-East plan and have more than one child and what could be the possible implications of these large families on both the children’s educational outcomes and parents’ economic situation. Bucharest, on the other hand, as the city capital, has a higher GDP per capita than the national average, with an economic structure based on services and a population twice as educated and wealthy (Voicu 2008). Individuals living in this region might display modern family formation patterns, either postponing the second child or adjusting their family size to just one child. No other significant regional differences in fertility intentions have been found.

Overall, the papers’ results are largely consistent with Klobas and Ajzen’s (2015) findings underlining the role of the social pressure and the positive attitudes towards childbearing in planning the first child. Focusing only on Romania, our paper investigates more thoroughly how people decide to have the first and second child and examines the role of the objective measures of control. Moreover, we included a larger set of background factors compared to Klobas and Ajzen (2015), who studied only age, education and partnership status. Additionally, by zooming in on the regional context to study childbearing intentions, we contribute to the empirical validation of the theoretical model in Romania.

The main limitation of this study lies in its cross-sectional nature; we cannot speak about a causal influence of the socio-psychological factors on childbearing intentions. The paper can be viewed as a first broad-brush approach to the correlates of the childbearing intentions in the traditional family context of Romania. With the possible collection of a new wave of the GGS program, which is about to become a European infrastructure project according to the next European Strategy Forum on Research Infrastructures (ESFRI) roadmap (Dușa et al. 2014), this study could serve as a starting point for further research on the determinants of fertility in Romania.

## Abbreviations

AIC: Akaike Information Criteria
GGS: Generation and Gender Survey
KMO: Kaiser-Meyer-Olkin measure of sampling adequacy
LAT: Living apart together
PAF: Principal axis factoring
PBC: Perceived behavioural control/perceived control
RMSR: Root mean square of the residuals
SDT: Second demographic transition
SN: Subjective norms
TFR: Total fertility rate
TPB: Theory of Planned Behaviour

## Footnotes

1 The family and reproductive policy during the Communist regime distinguishes Romania from the other ex-socialist countries. In 1957, following the Soviet lead, abortion was legalised, which lowered the TFR to 1.9, one of the lowest levels in the world at that time, similar to Hungary and Japan (Rotariu 2006). Romania’s 1966 anti-abortion decree and the ban of contraceptives (Berelson 1979) triggered high fertility rates until 1989, when the Communist regime fell.

2 The development regions of Romania refer to the eight regional divisions created in Romania in 1998 to better co-ordinate regional development as the country progressed towards accession to the European Union. The development regions correspond to NUTS-2 level division in EU member states. However, Romania’s development regions do not actually have an administrative status and do not have a legislative or executive council or government. Rather, they serve a function to allocate the European Union funds for regional development, as well as for collection of regional statistics. They also co-ordinate a range of regional development projects and became members of the Committee of the Regions when Romania joined the EU on January 1, 2007.

3 The development regions only broadly match the different ‘cultural areas’ identified by more homogenous historical and cultural criteria (Sandu 1999, 2011a). Nonetheless, the GGS data only has information about the NUTS-2 level of geographical areas.

4 In this study, we consider only the respondent’s parity; that is, a woman or man is defined as childless person or a one-child parent independently of their partner’s number of children, which can differ from the respondent’s in the case of step-families. The literature on step-families underlines the role of the partner’s child on fertility intentions (Thomson 2004). However, we do not control for step-children because of the low percentage of respondents with children from previous partnerships in our sample (6.7%). Moreover, research on stepfamilies in Romania on the same GGS data underlines the low percentage of this type of family (5%) (Steinbach et al. 2016).

5 Listwise deletion is not a proper procedure when missing values are not at random (MNAR), which is the case for income: the respondents with higher or very low income are less likely to report income than the others. We treat the missing values in two stages. First, we identify the respondents who did not declare their average monthly income, but who declared the range to which their income belongs. For them, we impute the missing values for the average income with the median of the income range. Then, for the respondents who declared neither the average income nor its range, we replace the missing values of non-response cases (recipients) with values extracted from response cases (donors) that are similar to the recipient in terms of the available observed characteristics, such as education, sex, residential area, development regions, occupation, health status and employment status. The method of taking a similar unit is called the Nearest Neighbour (NN) imputation procedure, which involves matching the group with missing data as closely as possible to the group without the missing data to reduce as much bias as possible when the missing values are imputed from the ‘donors’. This method is a generalisation of the so-called ‘hot-deck’ imputation (Andridge and Little 2010; Bankier et al. 2000; D’Agostino and Rubin 2000; Rubin 1986); it preserves the univariate distribution of the data and is one of the most used imputation methods in social research (Enders 2010). The remaining non-response items are treated in the regression analysis in a listwise deletion fashion.

6 Even in the case of the dependent variables listwise deletion is not a proper procedure (Rubin 1986). For this reason, we imputed the item non-responses using the NN procedure described in the footnote n.5. Following one of the reviewers’ suggestion, we ran the same regression models by deleting the missing values of the dependent variables: neither the significance nor the direction of the coefficients changed.

7 For both the childless individuals and one-child parents, we considered the validity measures of factor analysis: findings with factor loadings greater than 0.5 and with communalities over 0.4 (Stevens 2012). The items of each scale are internally consistent as the measure of reliabilities of each factor is high: Cronbach alpha values for all factors are higher than 0.7 (see Table 1 and Table 2), suggesting a high average correlation among the variables in each factor. For both subsamples, items with complex loadings and low communality (less than 0.3) were excluded from the analysis.

8 According to the Akaike Information Criterion (AIC) and the likelihood ratio test for model selection reported at the bottom Table 4
